# Dumb and Lazy? A Comparison of Color Learning and Memory Retrieval in Drones and Workers of the Buff-Tailed Bumblebee, *Bombus terrestris*, by Means of PER Conditioning

**DOI:** 10.1371/journal.pone.0134248

**Published:** 2015-07-31

**Authors:** Leonie Lichtenstein, Frank M. J. Sommerlandt, Johannes Spaethe

**Affiliations:** Department of Behavioral Physiology and Sociobiology, University of Würzburg, Würzburg, Germany; University of Tours, FRANCE

## Abstract

More than 100 years ago, Karl von Frisch showed that honeybee workers learn and discriminate colors. Since then, many studies confirmed the color learning capabilities of females from various hymenopteran species. Yet, little is known about visual learning and memory in males despite the fact that in most bee species males must take care of their own needs and must find rewarding flowers to obtain food. Here we used the proboscis extension response (PER) paradigm to study the color learning capacities of workers and drones of the bumblebee, *Bombus terrestris*. Light stimuli were paired with sucrose reward delivered to the insects’ antennae and inducing a reflexive extension of the proboscis. We evaluated color learning (i.e. conditioned PER to color stimuli) in absolute and differential conditioning protocols and mid-term memory retention was measured two hours after conditioning. Different monochromatic light stimuli in combination with neutral density filters were used to ensure that the bumblebees could only use chromatic and not achromatic (e.g. brightness) information. Furthermore, we tested if bees were able to transfer the learned information from the PER conditioning to a novel discrimination task in a Y-maze. Both workers and drones were capable of learning and discriminating between monochromatic light stimuli and retrieved the learned stimulus after two hours. Drones performed as well as workers during conditioning and in the memory test, but failed in the transfer test in contrast to workers. Our data clearly show that bumblebees can learn to associate a color stimulus with a sugar reward in PER conditioning and that both workers and drones reach similar acquisition and mid-term retention performances. Additionally, we provide evidence that only workers transfer the learned information from a Pavlovian to an operant situation.

## Introduction

*“Long before starting to build their first queen cells*, *the worker-bees have constructed some drone cells*, *from which the first drones are due to emerge about the beginning of May—lazy*, *stupid*, *fat*, *and greedy”*, *according to the German poet Wilhelm Busch*. *Indeed they do not attempt to take any part in the collection of food*, *an activity for which they are not properly equipped by nature*, *anyhow*. *Most of them are too indolent even to help themselves to their own share of the hive’s food stores*, *leaving it to the worker-bees to feed them*. *The brain of the drone is smaller than that of both worker and queen—we are not left in any doubt as to the intellectual inferiority of the male in this case*. [1]

Research about sensory and cognitive capabilities in eusocial bees, such as bumblebees and honeybees, has almost exclusively focused on workers, since they possess a wide repertory of colony-related behaviors like brood caring, cell building and cleaning, and foraging for pollen and nectar. In contrast, the role of drones within the colony as well as their cognitive capabilities have for centuries been only of minor scientific interest. Drones were often assumed to be dumb and lazy [1], a sentiment that dates back to Aristotle who noted that they are “devoid of sting, and lazy” (Hist. Anim. V, English translation by [2]). The majority of studies on drones have focused on mating tasks such as searching for and mating with queens (reviewed in [3]).

In the honeybee, *Apis mellifera*, newly emerged drones are fed by workers for the first few days until they are able to feed from honey combs within the hive [4, 5]. After about seven days drones start with orientation and mating flights [6, 7]. If they do not mate during their mating flight with a fertile young queen, they return to their colony or drift into neighboring hives to find shelter and to feed [7–9]. However, in most of the ca. 20,000 different bee species [10], mature males are not provided with food by the females, but have to forage by themselves (male traits in social insects are reviewed in [11]). In bumblebees, for example, drones stay after eclosion only for the first few days within the colony, before they fly out and never return. Thus, during most of their life they must find food and shelter by their own and thus must learn to recognize and discriminate rewarding flowers in order to collect pollen and nectar for their own needs [12]. Recently, it has been shown that males significantly contribute to the pollination of several plant species [13–15], and possess longer flower handling times and transfer larger amounts of pollen than females [13]. However, to what extent bumblebee drones can learn and memorize visual flower characteristics, has until present not been investigated.

Color conditioning experiments are well established with free-flying honeybee [16–20] and bumblebee foragers [21, 22], where individuals are rewarded with sucrose solution if they land on the intended color targets. However, this setup does not allow to completely control the environmental experience and stimulus perception of an individual. A more promising method for a quantitative evaluation of learning and memory in bees under controlled environmental conditions is the proboscis extension response (PER) assay ([23], for review see [24]). According to this method, bees are harnessed individually and learn to associate a conditioned stimulus (CS; e.g. novel odor or color) with an unconditioned stimulus (US; i.e. food taste). Naïve bees, for example, show the PER after the presentation of sucrose solution (US) to their antennae. After a few paired presentations of the CS and US, the CS alone provokes a conditioned response, the extension of the proboscis. PER conditioning is well established in honeybees [25, 26] and bumblebees [27–29] for which olfactory stimuli act as efficient CS. However, for several decades the PER assay failed in honeybees when visual stimuli were used as CS, except when the antennal flagellae were removed [23, 30–32]. Only recently, some groups successfully applied the PER assay using light as conditioned stimulus in intact workers of honeybees [33, 34] and bumblebees [35]. This might be explained by the fact, that in recent protocols the CS (color stimulus) was presented for a longer time (up to 15 s) than in earlier studies or studies using olfactory stimuli as CS (see also discussion).

In this study we performed visual PER conditioning in bumblebee drones and workers, and compared their acquisition and mid-term visual retention. Thus far, drones have been largely neglected in studies about learning and memory in bees (but see [36, 37]), even though drones of most social bee species undergo a different life history (mainly solitary and self-sustaining) than highly social and central place foraging workers. To address this issue, we conditioned the PER in intact bumblebee workers and drones using as CS different monochromatic light stimuli (435, 455, 488 and 528 nm) in combination with neutral density (ND) filters to vary light intensities. In this way, only chromatic cues were available as CS. Individuals were subjected to absolute (A+) and differential (A+ vs. B-) conditioning tasks (with A and B being the stimuli conditioned) and memory retention was tested two hours after the end of conditioning (mid-term memory; reviewed in [38]). Additionally, we studied if memories, established in the Pavlovian context of PER conditioning can be transferred to the operant free-moving context of a Y-maze in which bees were confronted to the formerly trained light stimuli. This transfer is possible after olfactory appetitive and aversive learning [39, 40] so that we aimed at determining if it is also possible in the visual domain.

## Material and Methods

### Preparing and Pre-Testing of Bumblebees

For all experiments we used workers and drones from *Bombus terrestris* colonies which were obtained from Koppert Biological Systems (Berkel en Rodenrijs, The Netherlands). The colonies were kept in a two chambered nest box (240x210x110 mm each chamber) at 25°C, 70% relative humidity and 12 h/12 h light/dark photoperiod. Each colony was provided with commercially available Apiinvert (a mixture of sucrose, fructose and glucose; Südzucker AG, Mannheim, Germany) and dried pollen ad libitum. Bees were randomly collected from their colony one day prior to conditioning. Individuals were chilled on ice and fixed in plastic tubes by means of paper clips and adhesive tape (as previously described by Sommerlandt et al. [29]). In this setup harnessed bumblebees could only move their head and the first pair of legs to facilitate perception of the US. Restrained bees were fed to saturation with a 30% sucrose solution (w/v) and placed over night in a dark climate cabinet (temperature: ~ 23°C; relative humidity: ~ 75%). Before the onset of the conditioning experiment, all bees were pre-tested for an intact PER by carefully touching the antennae with a toothpick soaked with 50% sucrose solution (w/v). For the conditioning experiments, we used only individuals that exhibited an intact PER during the pre-test.

### Stimuli Qualities and Experimental Setup

**Fig 1:**
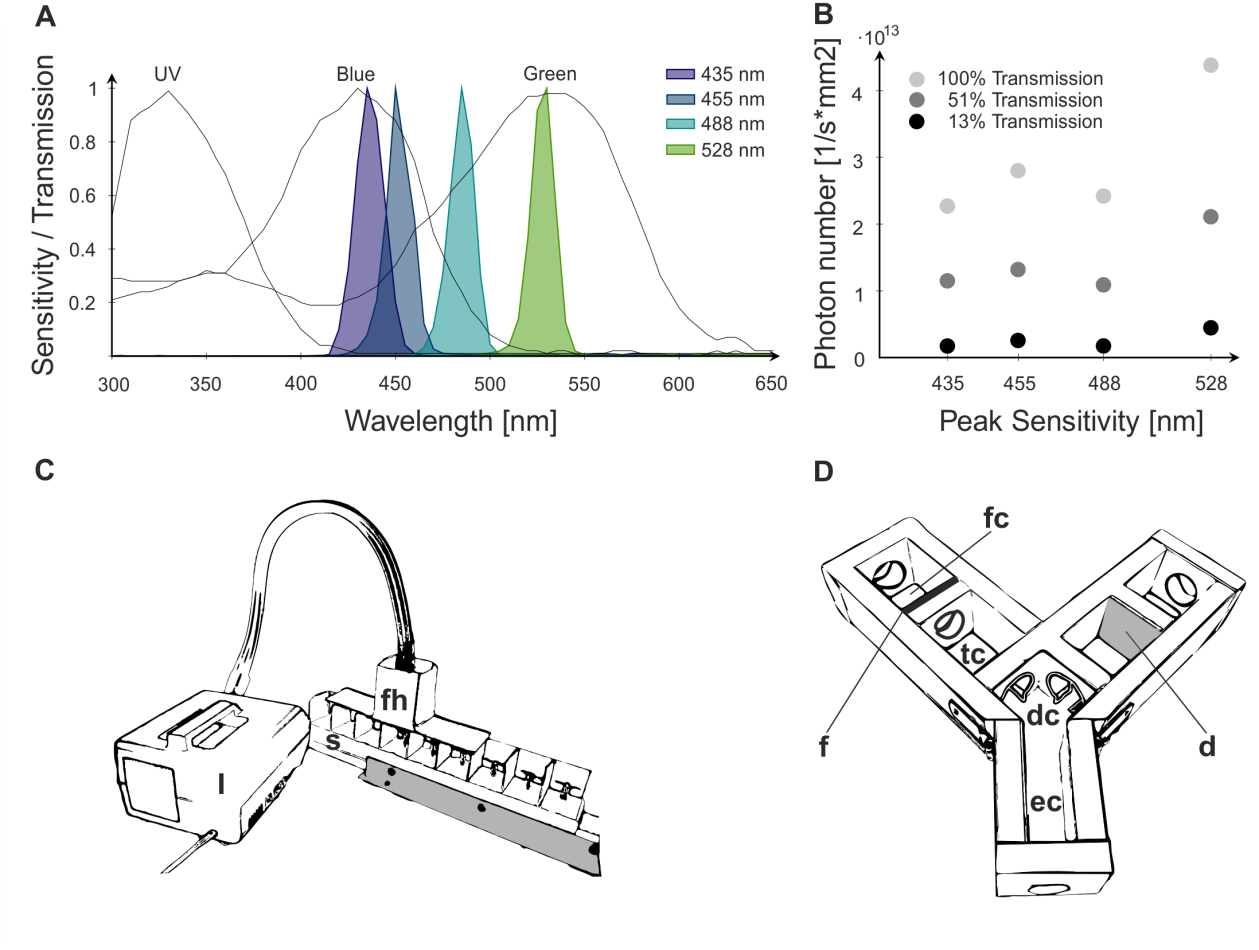
Stimuli qualities and experimental setup. A: Spectral sensitivity of the three photoreceptor types in *Bombus terrestris* (data obtained from [41]), overlaid by transmission of the four tested color filters (435 nm, 455 nm, 488 nm, and 528 nm). B: Intensities (photons per second and mm^2^) of the monochromatic light stimuli generated by means of different ND filters (13%, 51% and 100% transmission). C: Illustration of the set-up for visual PER conditioning. See text for description. fh, filter holder; s, movable sleigh. D: Y-maze set-up used for the transfer test after differential PER conditioning. The diffusor is omitted in the left arm to make the color filter (f) and the opening between the two chambers visible. See text for description. d, diffusor (parchment paper); fc, filter camber; f, color filter; tc, testing camber; ec, entrance chamber; dc, decision chamber.

For absolute and differential PER conditioning we used four different monochromatic light stimuli provided by different monochromatic filters (Schott & Gen, Jena, Germany) with absorption maxima at 435 nm, 455 nm, 488 nm and 528 nm and half band width of ca. 10 nm (Fig 1A). To prevent the bees from learning achromatic information such as brightness, we additionally used two ND filters with 13% and 51% transmission, respectively. Thus, each monochromatic light stimulus was presented at three different intensities (transmission 100%, 51% and 13%; Fig 1B). The conditioning setup consisted of a non-reflective gray acrylic movable sleigh with nine individual chambers (50 mm x 60 mm x 50 mm), a filter holder, which housed the color filters and ND filters, and a cold light lamp (Fig 1C). The filter holder could be placed above each chamber in which an individual bee was placed. Bees trained with differential conditioning were subsequently tested for a possible information transfer in a Y-maze made from plywood (Fig 1D). The Y-maze consisted of an entrance chamber (100 mm x 50 mm), in which the bees were released, followed by a decision chamber, in which the bees could choose to enter either one of the illuminated arms. The arms of the Y-maze were 200 mm long and 50 mm high, and arranged perpendicularly. Each arm was divided into a test chamber (tc; 100x50x50 mm; Fig 1D) and a filter chamber (fc; 80x50x50 mm; Fig 1D), and both chambers were connected via a circular opening, where the color filters were attached to. A bifurcated light guide attached to a cold-light lamp illuminated each of the filter chambers from the back side. In each arm, a color filter was attached at one side of the circular opening (f; Fig 1D), and a diffusor (parchment paper, d) at the other side to scatter the light which entered the test chamber. The light intensities for all tested colors were leveled by means of ND-filters. The setup was placed on a rectangular black cardboard, which was regularly replaced to exclude olfactory cues left by the walking bees, and covered with a Perspex plate. All experiments were conducted under red light conditions.

### PER Conditioning Protocol

The conditioning protocol was adapted from Riveros and Gronenberg [35] who showed for the first time that restrained bumblebees with intact antennae can be conditioned with light as CS using the PER paradigm. The training procedure started when the filter holder was placed on top of the chamber containing the first harnessed bee. Each individual was allowed to become accustomed for 10 s to the given situation. Afterwards the light stimulus was switched on for 12 s. 6 s after stimulus onset the bee was rewarded with 50% sugar solution presented on a tooth pick for 3 s. Following the offset of the light stimulus each bee had another 10 s rest before the sleigh was moved and the next bee was positioned under the filter holder. Although Riveros and Gronenberg [35] obtained high learning levels with their conditioning protocol, the 3s overhang of the CS after the US, has ended added a backward component to the conditioning procedure, which might have generated an inhibitory learning effect [42]. To test whether the prolonged CS affects the forward relationship between the light stimulus and the sucrose solution, we performed a control experiment with 528 nm as CS+ where we compared male and worker bees trained with the protocol mentioned above and a slightly modified protocol with no backward component, so that the CS and US ended at the same time (and thus comprised only a forward component, the procedure which is commonly used in Pavlovian conditioning).

In all conditioning experiments we used an inter-trial interval (ITI) of 8 min. A bee that responded with extending its proboscis during the first 6 s of stimulus exposure was scored as 1, whereas a bee that responded only to the sucrose reward or did not respond at all was scored as 0. Bees that showed no response to sucrose in more than four US presentations were excluded from further analysis.

### Absolute Color Conditioning

During absolute PER conditioning the bumblebees had to associate only one monochromatic light stimulus with a sugar reward. We trained two groups of bees: a test group and a control group. Within the test group each bee was trained over 10 trials, and the light stimulus (CS) and the sugar reward (US) were always presented simultaneously (paired group). Within the control group the light stimulus and the sugar reward were presented separately in different trials (unpaired group). Hence, each bee of the unpaired group had to complete 20 trials: 10 trials only with the light stimulus and 10 trials only with the sugar reward, in a randomized order. Using this protocol, the unpaired group received twice as many trials as the paired group (10 vs. 20 trials) which might led to a fatigue of animals in the control group (for a detailed discussion see [24]).

### Differential Color Conditioning

During differential PER conditioning the bees had to discriminate between a rewarded (CS+) and an unrewarded (CS-) light stimulus. Each bumblebee was trained over 18 trials (9 CS+ and 9 CS-) in a randomized order. To prevent bees from learning achromatic cues, we presented each light stimulus at three different intensities (transmission: 13%, 51% and 100%; Fig 1B).

### Mid-Term Memory Test

To assess mid-term memory retention of conditioned bumblebees, all individuals trained in absolute and differential conditioning were tested for memory retention two hours after the end of conditioning. To reactivate the bees and exclude individuals that do not react at all, bumblebees were tested for an intact PER just before re-testing. To exclude that the application of the US prior to the CS leads to an unspecific sensitization, bees that underwent absolute conditioning were confronted with the conditioned stimulus (CS) and with a novel color (NCol, as control) to test their response specificity. During stimulus presentation individuals were not rewarded and the succession of CS and NCol was randomized. When bees were trained to 435 nm, 455 nm and 488 nm, in each case the 528 nm light was used as NCol. For bees that were previously trained to 528 nm, NCol was 435 nm. In case of differential conditioning, we presented first the CS- and afterwards the CS+. At the end of the training protocol the CS+ was rewarded to avoid extinction learning prior to the subsequent Y-maze experiments.

### Y-Maze Transfer Test

To test whether the bumblebees can transfer the learned Pavlovian association from the PER conditioning to a novel operant free-moving context, we tested bees after differential conditioning and the two hour memory test in a Y-maze. All bees were chilled on ice until they calmed down (bees still showed slow movements of their antennae and first leg pair) and carefully released from their holders. They were then individually placed in the Y-maze and observed for 180 s following a protocol modified after Carcaud et al. [39]. A decision was recorded when the bee entered one of the illuminated test chambers within 180 s after release. Since chilling can have amnestic effects on olfactory memory [43] we performed control experiments (S1 Fig) were the bees were released from the holder and transferred to the Y-maze without chilling.

We used a custom-made computer program (YMaze, version 1.1) to document the first decision and the length of stay in any of the arms for each bee.

### Statistical Analysis

Statistics for the acquisition curves were done on the basis of an individual’s number of responses towards the light stimulus (depending on the number of trials between 0 and 9 in absolute, and between 0 and 8 in differential conditioning). In absolute conditioning, learning performance of paired and unpaired groups was compared using Mann-Whitney U test. Kruskal-Wallis test was used to compare the learning performance of all four monochromatic light stimuli. Memory retrieval was calculated with χ^2^ test statistics (fourfold table). In differential conditioning, learning performance within treatment groups was compared using Wilcoxon test and among groups using Mann-Whitney U test. Mann-Whitney U test was also applied to compare the discrimination index as a measure of performance [29, 44] between sexes and control experiments. Memory retrieval was calculated using χ^2^ test. First choice performance in the Y-maze transfer test was compared to random choice (50%) using Pearson’s χ^2^ goodness-of-fit test and the duration of stay in each arm was compared using Wilcoxon test. All statistics were calculated with IBM SPSS Statistics (Version 20.0.0) software.

## Results

### Absolute Color Conditioning

**Fig 2:**
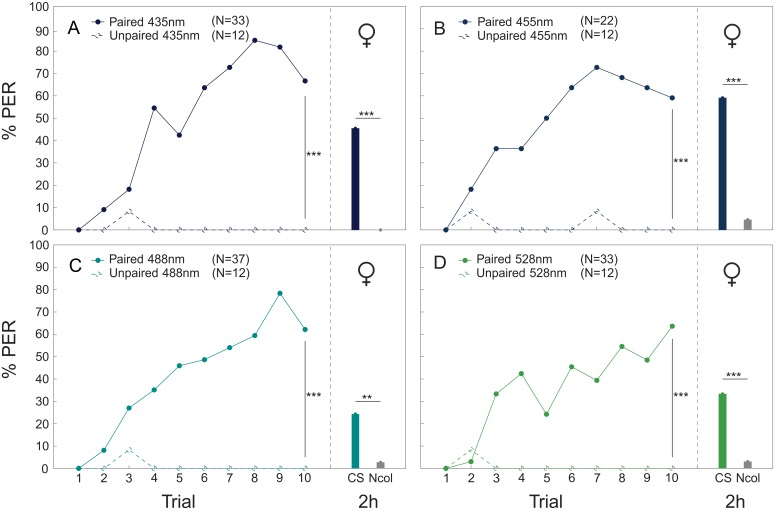
Absolute color conditioning and memory retrieval in workers. Acquisition curves (proportion of bees that responded to the tested color stimulus by extending the proboscis [% PER]) of workers during absolute conditioning of four different color stimuli (A: 435 nm, B: 455 nm, C: 488 nm and D: 528 nm). Workers were trained either with a paired (filled circles) or an unpaired (empty circles) presentation of CS and US. Memory retrieval was tested by presenting the CS (colored bar) and a novel color stimulus (NCol: gray bar) to the bees 2h after conditioning. *** P < 0.001; ** P < 0.01.

**Fig 3:**
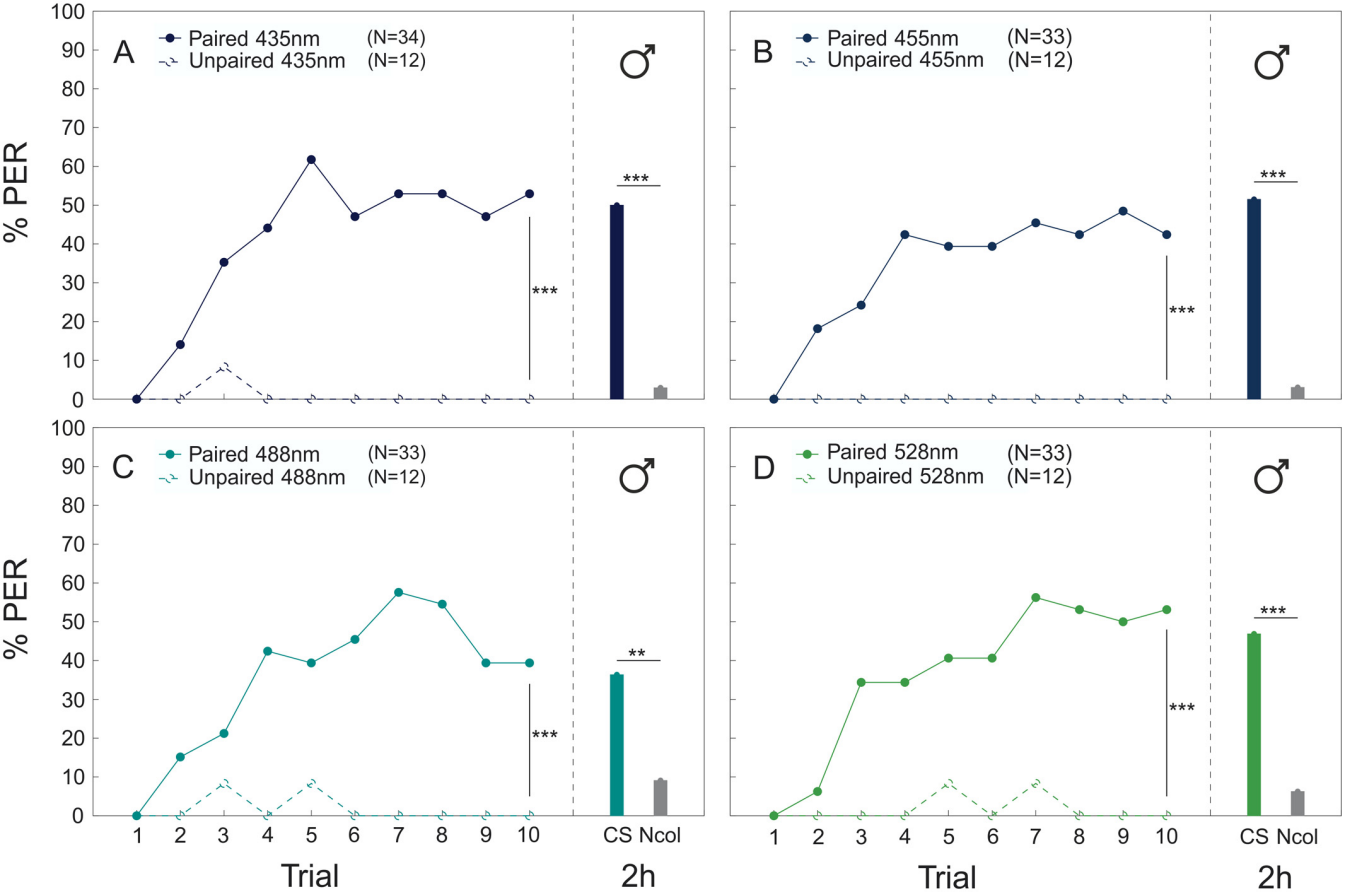
Absolute color conditioning and memory retrieval in drones. Acquisition curves (in % PER) of drones during absolute conditioning of four different color stimuli (A: 435 nm, B: 455 nm, C: 488 nm and D: 528 nm). Drones were trained either to a paired (filled circles) or an unpaired (empty circles) presentation of CS and US. Memory retrieval was tested by presenting the CS (colored bar) and a novel color stimulus (NCol: gray bar) to the bees 2h after conditioning. *** P < 0.001; ** P < 0.01.

When bees were trained to associate a sucrose reward with a monochromatic light stimulus (absolute conditioning), both workers and drones were able to build an association between CS and US after a paired presentation. For all colors, the paired groups in workers (435 nm: p<0.001, Z = -4.940; 455 nm: p<0.001, Z = -3.757; 488 nm: p<0.001, Z = -4.731; 528 nm: p<0.001, Z = -4.436; Fig 2) and drones (435 nm: p<0.001, Z = -3.636; 455 nm: p<0.001, Z = -3.514; 488 nm: p<0.001, Z = -3.702; 528 nm: p<0.001, Z = -3.305; Fig 3) performed significantly better than individuals trained with an unpaired presentation of the stimuli. No differences were found among color stimuli (workers: n.s., χ^2^ = 4,465; drones: n.s., χ^2^ = 0,673) or between sexes (n.s., Z = -1.798). In the mid-term memory test two hours after end of conditioning, workers (435 nm: p<0.001, χ^2^ = -19.412; 455 nm: p<0.001, χ^2^ = -15.086; 488 nm: p<0.001, χ^2^ = -7.400; 528 nm: p<0.001, χ^2^ = -10.185; Fig 2) and drones (435 nm: p<0.001, χ^2^ = -19.342; 455 nm: p<0.001, χ^2^ = -19.556; 488 nm: p = 0.008, χ^2^ = -6.988; 528 nm: p<0.001, χ^2^ = -13.537; Fig 3) responded significantly more often to the conditioned color stimulus than to a novel test color, which also indicates that activating the bees with sucrose solution before presenting the CS did not affect choice specificity.

**Fig 4:**
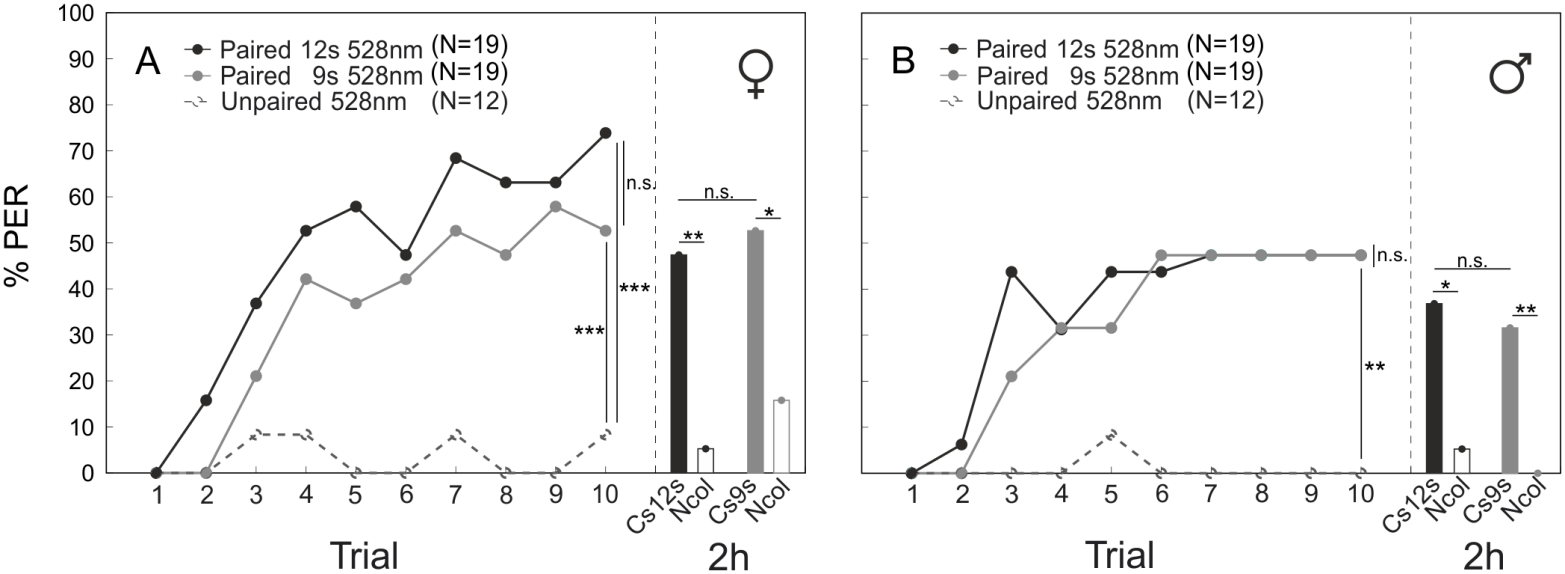
Impact of the conditioning protocol on performance in absolute color conditioning and memory retrieval. Learning curves of (A) workers and (B) drones during absolute conditioning. Bees were trained either with a paired (filled circles) or an unpaired (open circles) presentation of CS and US. Two groups of bees of each sex were trained with different conditioning protocols of the paired CS-US presentation: one group (*Paired 12* s) was presented in each trial with 12 s of CS, and 3 s of US 6 s after CS onset, which led to a 3s CS overhang after end of US; a second group (*Paired 9s*) received 9 s of CS and 3 s of US 6 s after CS onset. In the latter group, CS and US terminated simultaneously. Memory retrieval was tested by presenting the CS and a novel color stimulus (NCol) to the bees 2h after end of conditioning. **A**, *Paired 12 s/Unpaired*: MWU, p<0.001, Z = -3.587; *Paired 9 s/Unpaired*: MWU, p<0.001, Z = 3.587; *CS12 s/Ncol*: p = 0.003, chi^2^ = 8.686; *CS9 s/Ncol*: p = 0.017, chi^2^ = 5.729). **B**, *Paired 12 s/Unpaired*: MWU, p = 0.009, Z = -2.612; *Paired 9 s/Unpaired*: MWU, p = 0.009, Z = 2.612; *CS12 s/Ncol*: p = 0.017, chi^2^ = 5.700; *CS9 s/Ncol*: p = 0.008, chi^2^ = 7.125). *** P < 0.001; ** P < 0.01.

Since our protocol might have induced an inhibitory effect on learning performance due to the 3 s overhang of the CS after the end of the US presentation, we also tested an additional group of males and workers presenting the 528 nm light as CS+ but omitted the 3 s overhang. No significant difference was found in the learning performance (workers: n.s., Z = 1.192; drones: n.s., Z = -0.264) and mid-term retention (workers: n.s., chi^2^ = 0.105; drones: n.s., chi^2^ = 0.117) between both protocols and both sexes (Fig 4), indicating that the prolonged CS presentation did not induce any inhibitory effect on learning.

### Differential Color Conditioning

**Fig 5:**
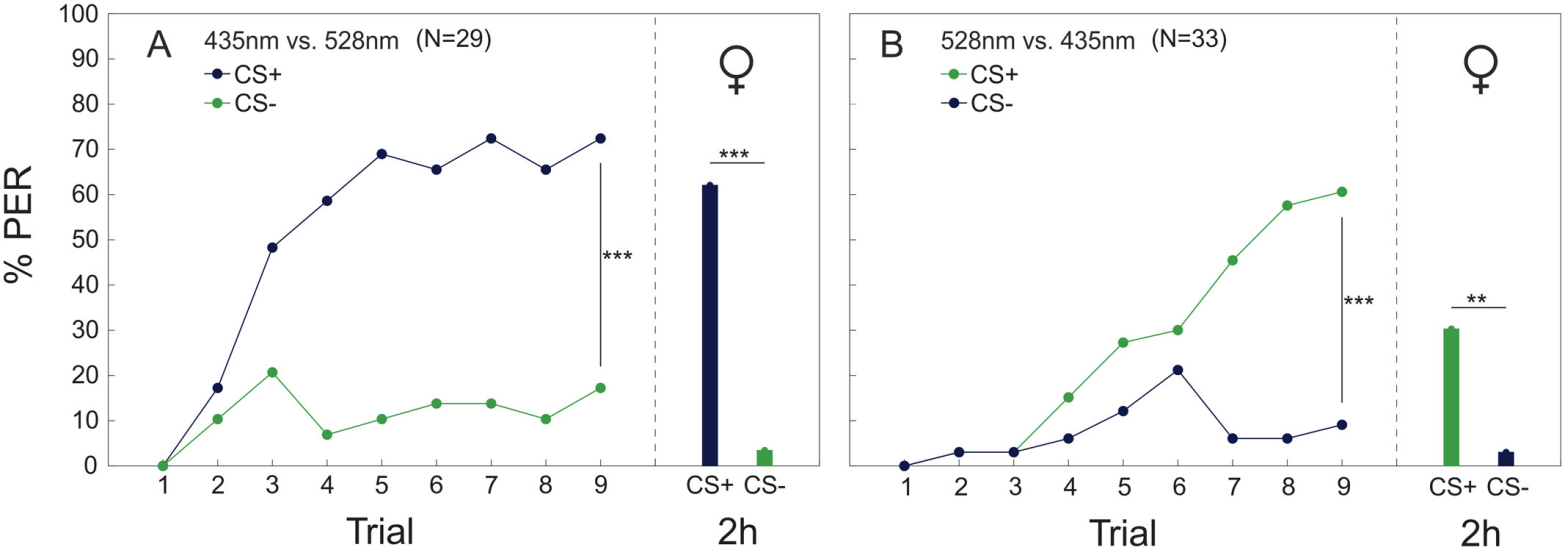
Differential color conditioning and memory retrieval in workers. Workers were trained by differential conditioning to discriminate between the color stimulus pair 435 nm and 528 nm (_Δ_λ = 93 nm). (A) 435 nm was used as the rewarded color stimulus (CS+) and 528 nm as the unrewarded color stimulus (CS), or (B) vice versa. Memory retrieval was tested by presenting the CS+ and the CS- to the bees 2 h after end of conditioning. *** P < 0.001; ** P < 0.01.

**Fig 6:**
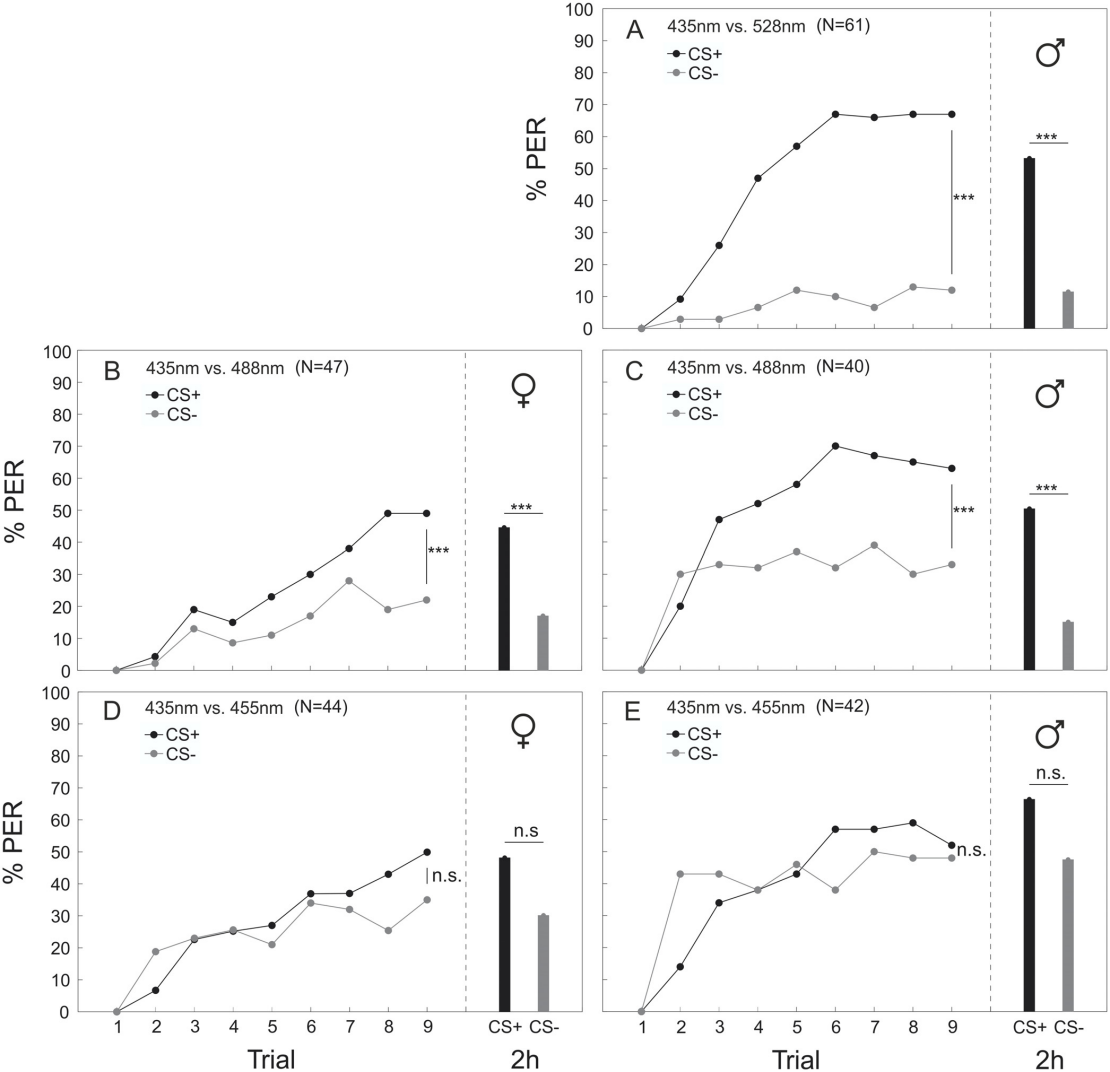
Differential color conditioning and memory retrieval in workers and drones. Three different monochromatic color stimuli combinations (435/528 nm; 435/488 nm and 435/455 nm) with different wavelength distances (93 nm; 53 nm and 20 nm) between stimuli were tested. Bumblebees were trained to discriminate the rewarded (CS+) and the unrewarded color stimulus (CS-). Each color stimulus combination was tested reciprocally. For the memory retrieval test the rewarded color stimulus (CS+: black bar) and the unrewarded color stimulus (CS-: gray bar) were presented to the bees 2 h after end of conditioning. Since no effects of asymmetrical discrimination between the two colors of each combination was found (except for 435/528 nm in workers, see Fig 5), data were pooled. *** P < 0.001; n.s.: not significant.

Bumblebees were able to discriminate different monochromatic light stimuli with large wavelength differences (Figs 5 and 6). Workers (435 nm vs. 528 nm: p<0.001, Z = -6.318; 435 nm vs. 488 nm: p = 0.001, Z = -3.306; Figs 5 and 6B) and drones (435 nm vs. 528 nm: p<0.001, Z = -6.092; 435 nm vs. 488 nm: p<0.001, Z = -4.175; Fig 6A and 6C) could significantly discriminate between CS+ (conditioned stimulus) and CS- (unconditioned stimulus) when the wavelength difference of the stimuli was 93 nm and 53 nm, respectively, irrespective of which wavelength was the rewarded or unrewarded stimulus. However, for the largest color distance, workers performed significantly better when 435 nm was the rewarded stimulus, compared to the case when 528 nm was rewarded (p<0.001, Z = -3.302; Fig 5). No such asymmetry was found in workers for the other two combinations (435 nm vs. 488 nm/455 nm; Fig 6B and 6D) or in any combination tested in drones (Fig 6A, 6C, 6E), so that data of counter experiments were pooled for statistical analysis (Fig 6). However, both workers (435 nm vs. 455 nm: n.s., Z = -1.837) and drones (435 nm vs. 455 nm: n.s., Z = -0.044) failed to discriminate stimuli with a relatively small wavelength difference of 20 nm (Fig 6D and 6E). The memory test revealed that workers (CS+_435_ vs. CS-_528_: p<0.001, χ^2^ = 22.621; CS+_528_ vs. CS-_435_: p = 0.003, χ^2^ = 8.836; Fig 5; 435 nm vs. 488 nm: p = 0.004, χ^2^ = 8.428; Fig 6B) and drones (435 nm vs. 528 nm: p<0.001, χ^2^ = 36.219; 435 nm vs. 488 nm: p = 0.001, χ^2^ = 11.168; Fig 6A and 6C) were able to recall the learned information two hours after conditioning. Both sexes failed the memory test for the smallest wavelength difference between 435 nm and 455 nm after two hours (workers: n.s., χ^2^ = 1.667; drones: n.s., χ^2^ = 3.048; Fig 6D and 6E). For the combinations 435 nm vs. 488 nm and 435 nm vs. 455 nm we also compared the discrimination index [28] between males and workers. No significant differences were found (435 nm/488 nm: n.s., Z = -1.315; 435 nm/455 nm: n.s., Z = -1.357), indicating that both sexes could discriminate the light stimuli equally well.

### Transfer Test to Y-Maze

**Fig 7:**
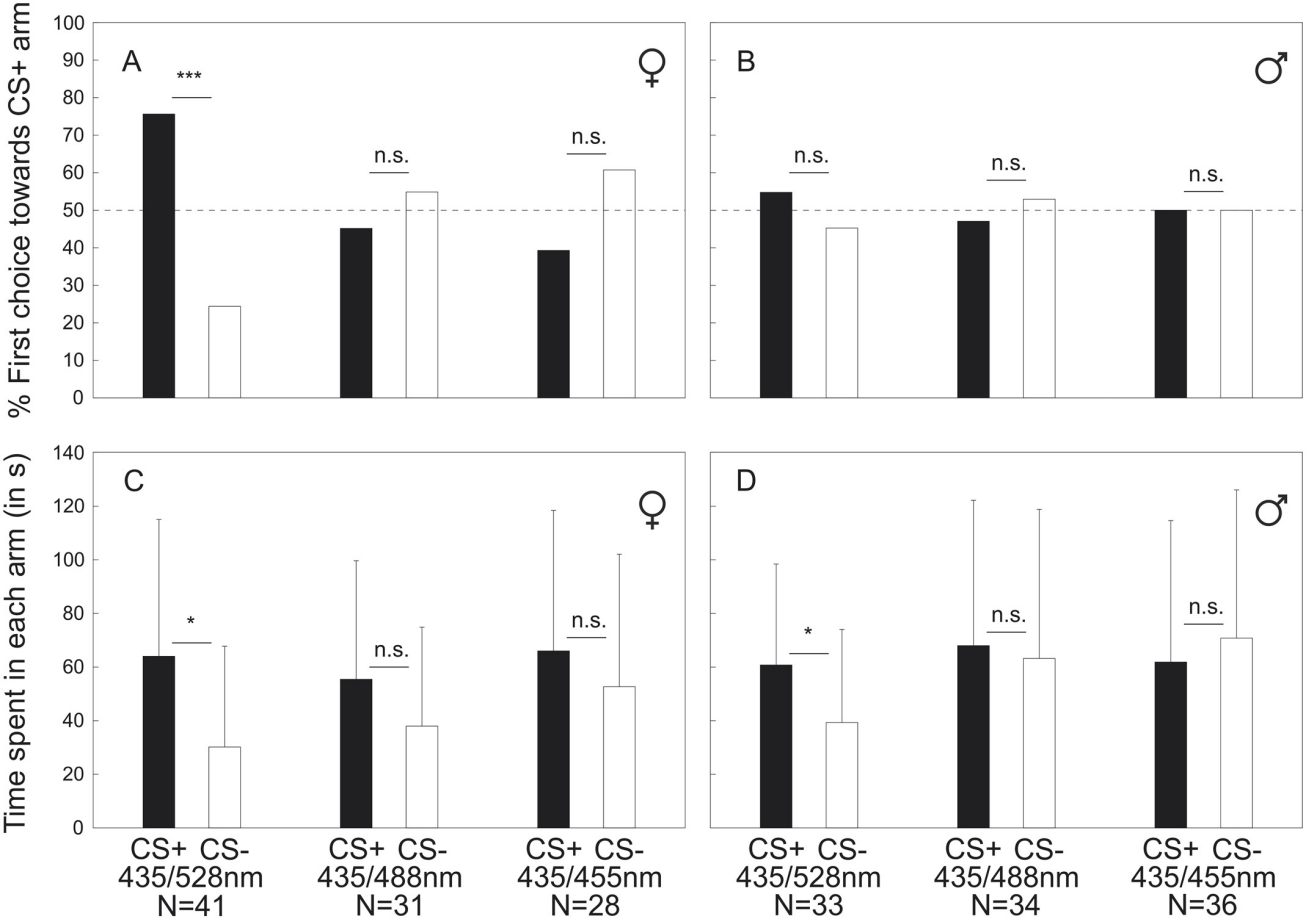
Information transfer after PER conditioning to Y-maze. Workers (A; C) and drones (B; D) were tested 2 h after end of conditioning for transfer of the learned color information to a novel operant context. Proportion of first choice of the bumblebees towards the CS+ arm (A: workers; B: drones) for three different color combinations (435/528 nm; 435/488 nm and 435/455 nm) and time (C: workers; D: drones) spent in each arm (in seconds). Since there were no significant differences within the respective color combination regarding the rewarded stimulus during the first choice towards the CS+ arm and time spent in each arm, all data were pooled for each tested color combination. *** P < 0.001; * P < 0.05; n.s.: not significant.

Individuals were tested for a transfer of the learned Pavlovian association to a novel operant free-moving situation in a Y-maze. Immediately after testing for memory retrieval, bees were confronted in the Y-maze with the same set of stimuli (CS+ and CS-) as they had experienced during differential PER conditioning. We recorded the first choice of a bee’s movement towards one of the two presented monochromatic light stimuli (Fig 7A and 7B). Additionally, we measured the time an individual spent in the respective arms during the first three minutes (Fig 7C and 7D). Workers chose significantly more often the arm with the previously learned color when the wavelength difference was largest (435 nm vs. 528 nm: p<0.001, χ^2^ = 10.756), but showed no preference when the wavelength differences were smaller (435 nm vs. 488 nm: n.s, χ^2^ = 0.290; 435 nm vs. 455 nm: n.s., χ^2^ = 1.286; Fig 7A). In contrast, drones showed no preference in their first decision when confronted with the previously rewarded color in the PER experiment, regardless of the combination of stimuli (435 nm vs. 528 nm: n.s., χ^2^ = 0.381; 435 nm vs. 488 nm: n.s, χ^2^ = 0.732; 435 nm vs. 455 nm: n.s., χ^2^<0.001; Fig 7B). When comparing the time the bees spent in both arms of the Y-maze, workers (p = 0.021, Z = -2.313) and drones (p = 0.018, Z = -2.357) stayed significantly longer in the arm in which the previously rewarded color was presented when the wavelength difference was largest (435 nm vs. 528 nm: 92 nm) (Fig 7C and 7D). For smaller wavelength differences, no preferences were observed for workers (435 nm vs. 488 nm: n.s., Z = -0.813; 435 nm vs. 455 nm: n.s., Z = -0.49; Fig 7C) or drones (435 nm vs. 488 nm: n.s., Z = -0.103; 435 nm vs. 455 nm: n.s., Z = -0.393; Fig 7D).

To exclude that chilling the bees before they were transferred to the y-maze interact with memory retrieval, we tested an additional group of workers which were not cooled when release from the fixation. No significant differences were found for the first choice and the time spend in each arm between groups (for statistics see supporting information, S1 Fig).

## Discussion

In the present study we provide evidence that (i) intact drones and workers of *Bombus terrestris* perform equally well in learning and discriminating monochromatic lights based only on chromatic information, (ii) workers but not drones show an asymmetric learning performance when they must discriminate between blue and green light, and (iii) workers and drones differ in their capability to transfer conditioned chromatic information acquired in a Pavlovian context to a novel operant free-moving situation.

Our data add to the recent findings that harnessed bees can be conditioned to monochromatic light stimuli in absolute and differential conditioning tasks without removing their antennae which is in contrast to some earlier studies. A possible explanation for the discrepancy is the prolonged presentation of the light stimulus, which was between 12 and 15 s in the studies where conditioning was successful ([34, 35], present study) but only 7 s where intact bees could not learn [30, 31].

In absolute conditioning, bumblebee workers as well as drones were able to learn all tested monochromatic lights (435, 455, 488, 528 nm). This is partially in line with findings from Africanized honeybees (AHB), where bees showed comparable acquisition curves when using light stimuli in the human-blue range of the chromatic spectrum [34]. In contrast, AHB performed much poorer compared to our bumblebees when they were conditioned to green light (520 nm) [34]. In our study, the learning performances of the bumblebees did not differ among the different tested stimuli-wavelengths. Another difference between both species refers to the memory test, where bumblebees were able to retrieve the learned color and successfully discriminate it from a novel color. AHB, in contrast, exhibited an overall poor memory retrieval [34]. However, this statement should be treated with caution since we tested mid-term memory (2h), whereas AHB workers were tested after 24 h (early long-term memory; [34]). Neither of these memories rely on protein synthesis (reviewed by Menzel [38]) and, as reported for olfactory conditioning, memory performance on population level does not differ between mid-term and early long-term memory [45].

To assess wavelength discrimination capabilities, we tested three pairs of monochromatic lights with decreasing wavelength differences (93 nm, 53 nm and 20 nm, respectively) in a differential conditioning paradigm. We showed that workers and drones were able to discriminate between different monochromatic light stimuli and retrieve the learned stimulus after two hours. These results coincide with findings by Riveros and Gronenberg [35] who reported that *Bombus impatiens* workers were able to discriminate color stimuli with a difference of 53 nm using differential conditioning. The minimal color distance that can be distinguished and learned by workers and drones in our study was between 20 and 53 nm, thus indicating a poorer color discrimination capability compared to free flying honeybees. In the early 1970’s, von Helversen first demonstrated that free flying honeybees were able to discriminate different monochromatic colors and found a discrimination threshold of 15 nm in our wavelength area [46]. The poorer discrimination abilities in our study might be attributed to the fixation of the bees, since not only harnessed bumblebees but also harnessed honeybees showed a worse discrimination performance [31]. Further explanation for poorer discrimination might be the type of training. In the PER setup, stimuli are presented successively, while in experiments with freely moving bees, target and distractor stimuli can be perceived simultaneously. It has been shown that the manner of stimuli presentation is essential for discrimination performance [21]. In contrast to successive presentation of the color stimuli, simultaneous presentation enables bees to choose the features which allow an easy discrimination between rewarded and non-rewarded stimulus. In short, honeybees [47] and bumblebees [21] perform significantly better when the test stimuli are presented at the same time.

Surprisingly, the behavior of males and workers differed when they had to discriminate between blue (435 nm) and green (528 nm) light. While workers and drones performed equally well when the blue light was presented as CS+ and green light as CS-, workers performed significantly poorer compared to drones in the reversed situation, i.e. when discriminating 528 nm as CS+ from 435 nm as CS-. This phenomenon might be explained by an intrinsic preference of workers for blue color stimuli [48, 49] that may bias performance and affect visual learning, as recently shown for honeybees [50]. For workers it might be of advantage to possess a color preference when leaving the hive on their first foraging flight. Previous studies have shown that bumblebee workers possess an innate preference for violet and blue [49, 51]. This innate preference coincides with findings that violet and blue (i.e. bee-blue and bee-UV-blue) flowers provide on average more nectar than flowers of any other color category [48]. Furthermore, bumblebee workers showed faster and higher learning acquisition rates for stimuli with shorter wavelengths [35, 51]. Bumblebee drones, in contrast, are not subjected to evolutionary pressure for efficient foraging, since they only need to obtain food for themselves and do not forage for the colony, i.e. they have no impact on the colony’s fitness. Thus, an innate color preference might not contribute to the drones’ fitness. However, the proximate reasons for the sex-dependent differences we observed needs to be investigated.

In all previous studies testing color discrimination by means of PER, chromatic LED lights were used and adjusted to equal brightness (measured as photon numbers) [33–35] to prevent the bees from learning brightness differences between stimuli. However, photoreceptors adapt to the background intensity and hence the sensitivity of the photoreceptors can significantly differ [46, 52], causing different receptor excitations despite identical stimulus intensities. In his seminal work, von Helversen [46] demonstrated that honeybees are >10 times more sensitive for UV than for green light [46]. This implies that a standardized brightness (adjusted to equal number of photons) might still lead to an unequal excitation of different photoreceptors and hence a different perception level. To address this issue and to provide a “reliable method of demonstrating color vision” [53], we used monochromatic filters in combination with different ND filters to prevent bees from learning receptor-specific excitation differences instead of chromatic differences.

Under natural conditions, foraging bees may profit from capabilities which allow them to transfer information gained in one context to a novel situation. Specific flower characteristics, for example, can be learned and associated with a nectar reward, although flowers never appear twice with exactly identical properties and under the same environmental conditions. Nevertheless, foragers must recognize flower types and thus generalize to a certain degree in order to optimize foraging rates. Moreover, information transfer is also necessary when information (e.g. flower specific odors) about profitable food sources is communicated inside the nest of social bees. Newly recruited foragers can learn the odor of recently collected nectar (provided by nestmates) and use this information on their own foraging flights (bumblebees: [54]; honeybees: [55]). When honeybees are exposed to odors during early adult stages or foraging flights, they later respond in PER conditioning more frequently to the experienced odor, but have difficulties associating a new odor with a sugar reward [56, 57]. Mc Cabe and Farina [58] reported a transfer of olfactory information in stingless bees where learning performance of *Melipona quadrifasciata* in olfactory PER conditioning was positively influenced by previous in-hive experiences of the learned odor. Moreover, honeybees were shown to transfer olfactory information acquired during PER conditioning to a novel operant choice situation in a Y-maze [39] or orientation flight [59]. In the present study we trained bumblebees to discriminate between pairs of color stimuli in the PER paradigm, before we tested them two hours later for information transfer in a Y-maze choice experiment. Bumblebee workers chose significantly more often the arm of the Y-maze where the previously rewarded color was presented and also spend more time in that arm. This was true for the stimuli pair with the largest wavelength difference (425 nm vs. 528 nm), but not for smaller color differences. Drones, in contrast, showed no preference towards the reinforced color target in their first decision, even when presenting the largest color difference. However, when testing the 425 nm / 528 nm pair, drones spent more time in the chamber illuminated by the previously rewarded color, compared to the unrewarded color. Drones in general were significantly faster in making their first decision compared to workers (34 seconds vs. 49 seconds; p<0.001, Z = -3.241, MWU test; data not shown). These differences could be based on a sex-specific difference in speed and accuracy trade-off, as shown by Chittka et al. [60] on the level of individual bumblebee workers. In general, bumblebee workers may need to transfer information about rewarding flowers (e.g. the floral scent) acquired in the hive from returning foragers to direct their own foraging behavior towards the advertised flower type ([54], for review see [61]). In contrast, drones do not rely on such information transfer abilities, since they do not communicate with other conspecifics (but see Grüter and Leadbeater [62] for potential information gain via social information).

The fact, that males possess similar learning skills compared to workers might be surprising at first glance, since at least in honeybees, drones have been believed to perform only simple, reflex-like behavior, such as feeding and mating [63]. These behaviors might be facilitated by a simple response to olfactory, visual or tactile key stimuli [64]. Workers, in contrast, show more complex social behaviors. As central place foragers, they must not only recognize, but also memorize locations and landmarks, as well as shapes, colors and odors of profitable food sources. However, honeybee drones are reported to successfully associate odor stimuli with food reward in PER conditioning [36, 65] and colored light stimuli with electric shocks in a free-moving avoidance assay [66]. Moreover, drones of *Bombus terrestris* have been successfully trained to learn olfactory stimuli using the PER paradigm and performed equally well in absolute and differential conditioning compared to workers [67]. In both, drones and workers, learning and memory formation is the product of the (central) nervous system and there is no reason to assume that the neuroanatomical structure is completely different between sexes and casts of the same species. Although sex-specific modifications may appear in the brain, and particular at the peripheral sensory level, which enables the bearer to become particular sensitive to distinct stimuli [68, 69], the neuronal processes connecting a stimulus (CS+) to the reward system (US circuit; [70]) probably constitute a basic feature of the nervous system of all bees and even insects.

## Conclusion

In the present study we could show that intact bumblebees are able to associate a sugar reward with a monochromatic light stimulus in an absolute and differential Pavlovian conditioning paradigm. In contrast to honeybees, bumblebees can be easily trained without antennal deprivation. We were able to establish a visual PER conditioning setup for bumblebee workers and drones, and found similar learning and memory performance in both sexes. Workers were also capable of transferring learned information to a new behavioral context. Due to their phylogenetic relationship to honeybees, their experimental robustness and their learning abilities, bumblebees provide a suitable model to study the neurobiological and molecular mechanisms underlying visual learning and memory formation by means of classical PER conditioning.

## Supporting Information

S1 Fig Effect of cooling on information transfer. Workers were trained in differential PER conditioning (A, B) and 2h later (after the mid-term memory test) tested for transfer of the learned color information to a novel operant free-moving situation (Y-maze; C, D). Two groups were tested: in one group bees were slightly chilled on ice right before their fixation was removed and they were transferred to the Y-maze, the other group of bees was not cooled at all. Discrimination index (n.s., Z = -0.228) and memory retrieval (response to CS+; n.s.; chi^2^ = 0.167), as well as performance in the Y-maze (first decision: n.s., chi^2^ = 0.038; duration of stay in the CS+ arm: n.s.; Z = -0.456) did not differ between treatment groups. From the results we can conclude, that slightly cooling on ice before the transfer test had no significant effect on bee’s choice behavior in the Y-maze.
(PDF)
