# Supplementary material for: Dumb and Lazy? A Comparison of Color Learning and Memory Retrieval in Drones and Workers of the Buff-Tailed Bumblebee, Bombus terrestris, by Means of PER Conditioning
Source: PLoS One. 2015 Jul 31;10(7):e0134248. doi: 10.1371/journal.pone.0134248 (PMC4521843; doi:10.1371/journal.pone.0134248)
Supplement: S1 Fig — Workers were trained in differential PER conditioning (A, B) and 2h later (after the mid-term memory test) tested for transfer of the learned color information to a novel operant free-moving situation (Y-maze; C, D). Two groups were tested: in one group bees were slightly chilled on ice right before their fixation was removed and they were transferred to the Y-maze, the other group of bees was not cooled at all. Discrimination index (n.s., Z = -0.228) and memory retrieval (response to CS+; n.s.; chi2 = 0.167), as well as performance in the Y-maze (first decision: n.s., chi2 = 0.038; duration of stay in the CS+ arm: n.s.; Z = -0.456) did not differ between treatment groups. From the results we can conclude, that slightly cooling on ice before the transfer test had no significant effect on bee’s choice behavior in the Y-maze. (PDF) [file pone.0134248.s001.pdf]

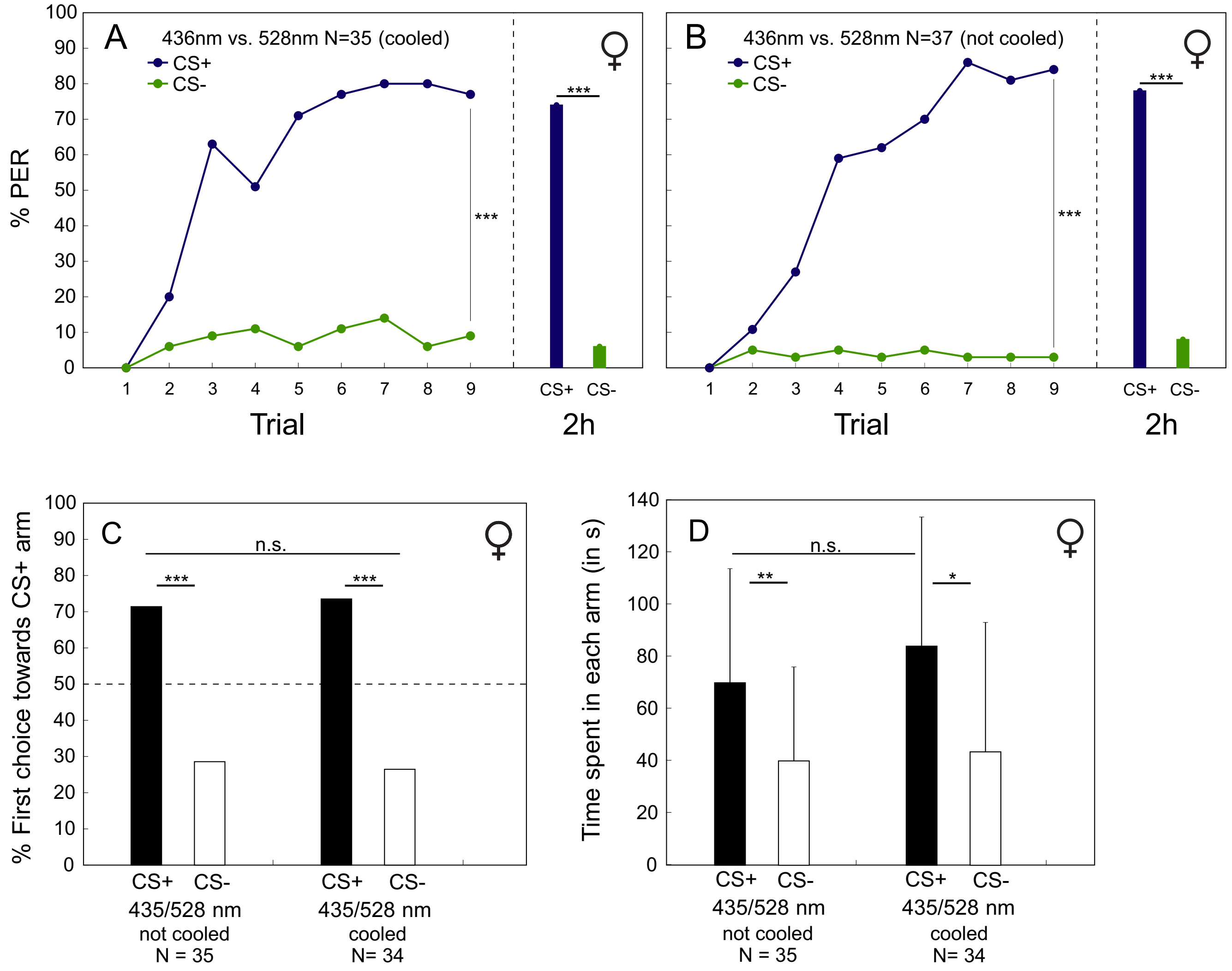

S1 Fig. Effect of cooling on information transfer.
